# Multisystem inflammatory syndrome in children (MIS-C) presenting with valvulitis, myocarditis, and QTc prolongation: A case report from Saudi Arabia

**DOI:** 10.1097/MD.0000000000043995

**Published:** 2025-08-29

**Authors:** Sultan Alharthi, Suzan Abdullah Jafri, Mazen A. Alzaedi, Nejood Metaeb Aljaed, Mohammed Mahmoud Eldoskey, Salma A.S. Abosabie, Mohammed Oshi, Sara A. Abosabie, Naglaa Mohamed Kamal

**Affiliations:** a Pediatric Department, Alhada Armed Forces Hospital, Taif, Kingdom of Saudi Arabia; b Pediatric Department, Prince Mansour Military Hospital, Taif, Kingdom of Saudi Arabia; c Faculty of Medicine, Julius-Maximilians-Universität Würzburg, Bavaria, Germany; d Pediatric Department, Gaafar Ibnauf Children’s Emergency Hospital, Khartoum, Sudan; e Faculty of Medicine, Charité – Universitätsmedizin Berlin, Berlin, Germany; f Kasralainy Faculty of Medicine, Cairo University, Cairo, Egypt.

**Keywords:** cardiac complications, COVID-19, MIS-C, myocarditis, pediatric case report, QTc prolongation, SARS-CoV-2, Saudi Arabia, valvulitis

## Abstract

**Rationale:**

This case report highlights the complex clinical course and successful multidisciplinary management of a pediatric patient with multisystem inflammatory syndrome in children (MIS-C), who posed clinical dilemma at presentation. It underscores the ongoing clinical relevance of MIS-C as a post-Coronavirus disease 2019 sequelae and emphasizes the importance of maintaining a high index of suspicion for MIS-C in pediatric differential diagnoses, especially when symptoms overlap with other common conditions.

**Patient concerns:**

An 11-year-old previously healthy Saudi girl presented with gastrointestinal symptoms initially suggestive of acute appendicitis. Her condition rapidly deteriorated with signs of cardiovascular compromise.

**Diagnoses:**

Surgical exploration confirmed a perforated appendix. Cardiac workup revealed elevated troponin levels, corrected QT interval prolongation (500 ms), ST-segment changes, and echocardiographic evidence of mitral and aortic regurgitation with reduced ejection fraction, leading to a diagnosis of MIS-C fulfilling both Centers for Disease Control and Prevention and World Health Organization criteria. Schwartz et al’s criteria are widely accepted for diagnosing long QT syndrome, which guided our interpretation of the corrected QT interval prolongation observed in this case. According to the Centers for Disease Control and Prevention, MIS-C is defined by a constellation of symptoms occurring in individuals under 21 years with recent severe acute respiratory syndrome coronavirus 2 infection or exposure.

**Interventions:**

Management included intravenous immunoglobulin, corticosteroids, inotropes, diuretics, aspirin, and broad-spectrum antibiotics, coordinated by a multidisciplinary care team.

**Outcomes:**

The patient experienced full cardiac recovery, confirmed through serial electrocardiogram and echocardiography over 1 year.

**Lessons:**

This case underscores the importance of recognizing MIS-C in children presenting with atypical symptoms such as abdominal pain. Timely diagnosis and early multidisciplinary intervention are essential to prevent serious cardiac complications.

## 1. Introduction

Coronavirus disease 2019 (COVID-19), identified as a novel disease caused by the severe acute respiratory syndrome coronavirus 2 (SARS-CoV-2) virus, has evolved into a global health crisis since its emergence in 2019. As of December 6, 2023, the World Health Organization (WHO) has documented over 772 million confirmed cases and nearly 7 million fatalities worldwide.^[1]^ Initial research from China, Italy, and the USA indicated that severe disease from COVID-19 among children was initially felt to be rare, though infection itself was relatively common, with incidences ranging from 1.2% to 2%, predominantly affecting adults.^[2,3]^ The clinical spectrum in children varies widely, from asymptomatic cases to severe illness.^[2,4]^ Common symptoms in the pediatric demographic include cough, fever, headache, sore throat, shortness of breath, myalgia, and gastrointestinal issues such as nausea, vomiting, abdominal pain, and diarrhea. Leukopenia, lymphopenia, and elevated inflammatory markers are among the most observed laboratory abnormalities, while renal, cutaneous, olfactory, ocular, and neurological symptoms are less frequently reported.^[2–4]^ Adult patients tend to experience more severe symptoms, including cardiac injury and inflammation, such as myocarditis.^[2]^ However, since spring 2020, a distinct clinical entity known as multisystem inflammatory syndrome in children (MIS-C) has been recognized and epidemiologically linked to COVID-19, manifesting with persistent fever, elevated inflammatory markers, acute cardiovascular distress, and gastrointestinal symptoms that sometimes resemble acute appendicitis.^[5–7]^

Some affected children have developed severe inflammatory conditions with symptoms akin to toxic shock syndrome or Kawasaki disease, including persistent fever, fatigue, diffuse rash, non-purulent conjunctivitis, gastrointestinal symptoms, and, in severe cases, hypotension and shock, necessitating intensive care.^[6,8]^ In the United States, the incidence of MIS-C among those infected with SARS-CoV-2 is estimated at 316 per million infections.^[3]^ Despite unknown prevalence rates, over 4018 cases have been reported in the USA alone.^[3]^

The Centers for Disease Control and Prevention (CDC) has established criteria for diagnosing MIS-C in individuals under 21 years, which include fever, evidence of inflammation, severe illness requiring hospitalization, involvement of 2 or more organ systems, and a positive test for SARS-CoV-2 infection or exposure to a confirmed case.^[9]^ Similar criteria have been released by the WHO emphasizing the need for a positive SARS-CoV-2 test result or exposure and the exclusion of other microbial causes of inflammation.^[1,9,10]^

Certainly, after being admitted to the hospital for MIS-C most patients are discharged, however, approximately 2% of patients with MIS-C die.^[9]^ It was found in MIS-C cases that the involvement of digestive system issues was about 70% of cases, skin rash 57% of cases and heart and blood vessel problem 52% of cases which can lead to shock.^[6]^ Cardiac manifestation of MIS-C vary but significantly impact patient outcomes. Myocardial dysfunction has been reported in about 75% of patients in some series, with ventricular dysfunction observed in 35 to 100% of MIS-C cases. The exact mechanisms behind myocardial dysfunction in MIS-C remain under investigation, but potential causes include acute myocarditis, hypoxic injury, ischemic injury due to cardiac microvascular damage, right heart strain, stress cardiomyopathy, and systemic inflammatory response syndrome.^[5,6,11]^ Coronary artery dilatation or aneurysm has been observed in 6% to 24% of patients, with some cases progressing to large or giant coronary artery aneurysms.^[5,9,12]^ Arrhythmias, including nonspecific electrocardiogram (ECG) anomalies, corrected QT interval (QTc) prolongation, premature atrial or ventricular beats, and even atrial fibrillation, have been noted in 7% to 60% of patients, highlighting the diverse and serious cardiac complications associated with MIS-C.^[5,9,13]^ Factors associated with severe outcomes in MIS-C have been extensively reviewed by Abrams et al.^[5]^

## 2. Case presentation

An 11-year-old previously healthy Saudi girl presented with a 2-day history of progressive abdominal pain and vomiting. The colicky pain, of moderate intensity, was localized centrally without radiation, accompanied by 4 episodes of yellowish, non-projectile vomitus without blood or mucus. She reported a subjective fever, which responded to antipyretics, along with mild constipation. Fifteen days prior, she had been in close contact with her father, who tested positive for COVID-19.

**Figure 1.**
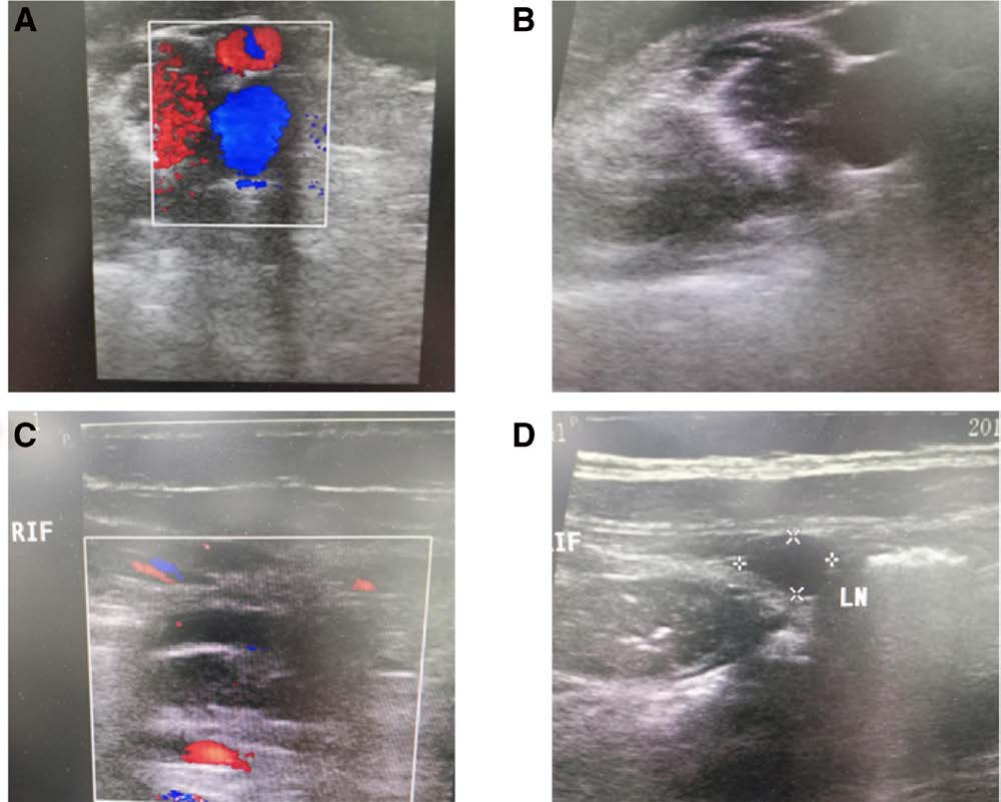
Abdominal ultrasound showing (A) noncompressible, blind-ended tubular structure in the right iliac fossa (suggestive of appendicitis). (B) Dilated bowel loop and localized fluid collection adjacent to the inflamed appendix. (C) Reactive mesenteric lymph nodes. (D) Features consistent with complicated appendicitis.

During her initial emergency room visit, gastritis was diagnosed, and she was treated with antiemetics and intravenous fluids before being discharged. However, her condition worsened, prompting another emergency room visit in the second day. At this time, the pain localized to the right iliac fossa, with tenderness, rebound, and a positive Psoas sign. Laboratory findings revealed leukocytosis with neutrophilia, elevated inflammatory markers, and slightly elevated creatinine levels; other parameters, including hemoglobin, platelets, liver function tests, and coagulation profiles, were within normal ranges. Abdominal ultrasound (Fig. 1) identified a noncompressible, blind-ended tubular structure in the right iliac fossa, measuring 0.8 cm in diameter, consistent with appendicitis.

The patient was admitted for surgical intervention and started on amoxicillin–clavulanic acid and acetaminophen. Surgical intervention was deferred at that point due to stable vital signs and absence of peritoneal signs, in line with initial conservative management for suspected uncomplicated appendicitis. However, the patient’s condition deteriorated rapidly thereafter, leading to perforation. Despite this, her condition deteriorated rapidly, presenting with fever (40 °C), agitation, pallor, bilious vomiting, tachypnea, tachycardia (170–190 bpm), hypotension (81/39 mm Hg), delayed capillary refill (>3 s), and cold extremities. After receiving 60 mL/kg of saline boluses, her hemodynamic instability persisted, necessitating transfer to the pediatric intensive care unit under suspicion of septic shock. At the pediatric intensive care unit, an additional 20 mL/kg fluid bolus was administered, along with epinephrine infusion (0.3 mcg/kg/min) and broad-spectrum antibiotics (vancomycin, meropenem, and metronidazole).

Surgical exploration revealed a ruptured retrocecal appendix with a small abscess, which was successfully managed. Postoperatively, the patient experienced persistent hypotension requiring norepinephrine and oxygen therapy. Follow-up investigations demonstrated transient leukocytosis and inflammatory marker fluctuations, with initial neutrophilia normalizing over time. Temporary abnormalities in liver and renal function tests, coagulation profiles, and bone markers were managed effectively, and blood cultures were negative.

The patient’s initial ECG showed a QT interval of 420 ms and an RR interval of 800 ms, yielding a calculated QTc of 500 ms using Bazett formula, which is considered prolonged for her age and sex. According to established pediatric references, the normal QTc in pre-adolescent females is ≤460 ms.^[6]^

**Figure 2.**
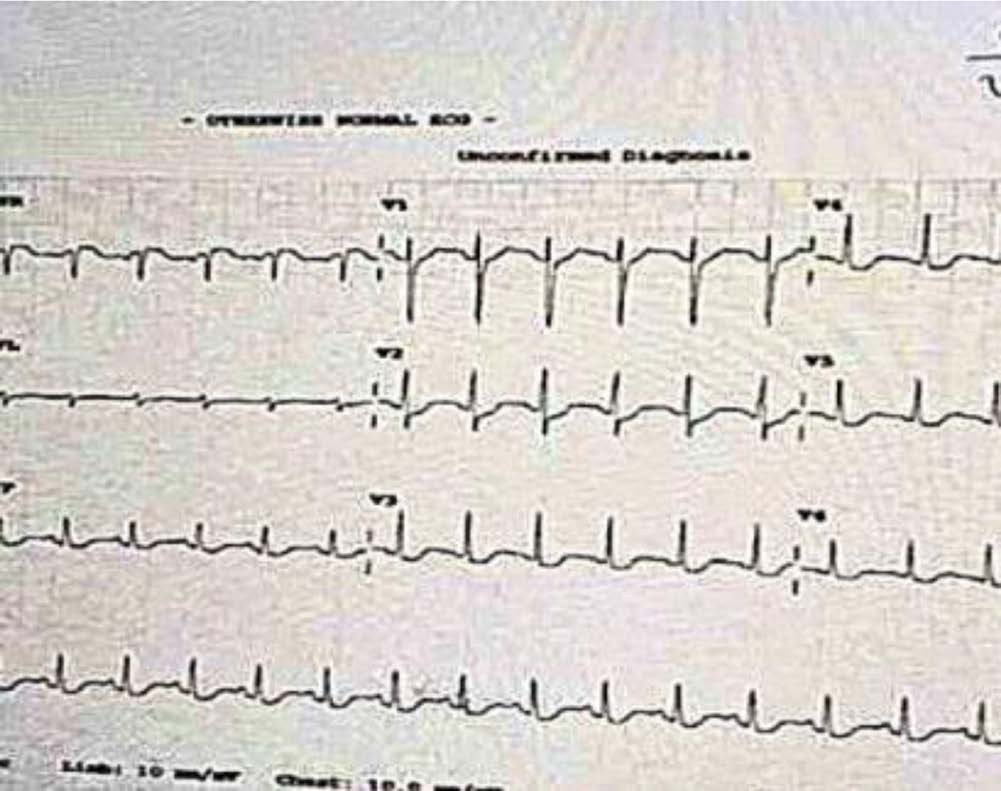
Initial ECG: mild ST segment depression and T-wave touching P-wave. ECG = electrocardiogram.

**Figure 3.**
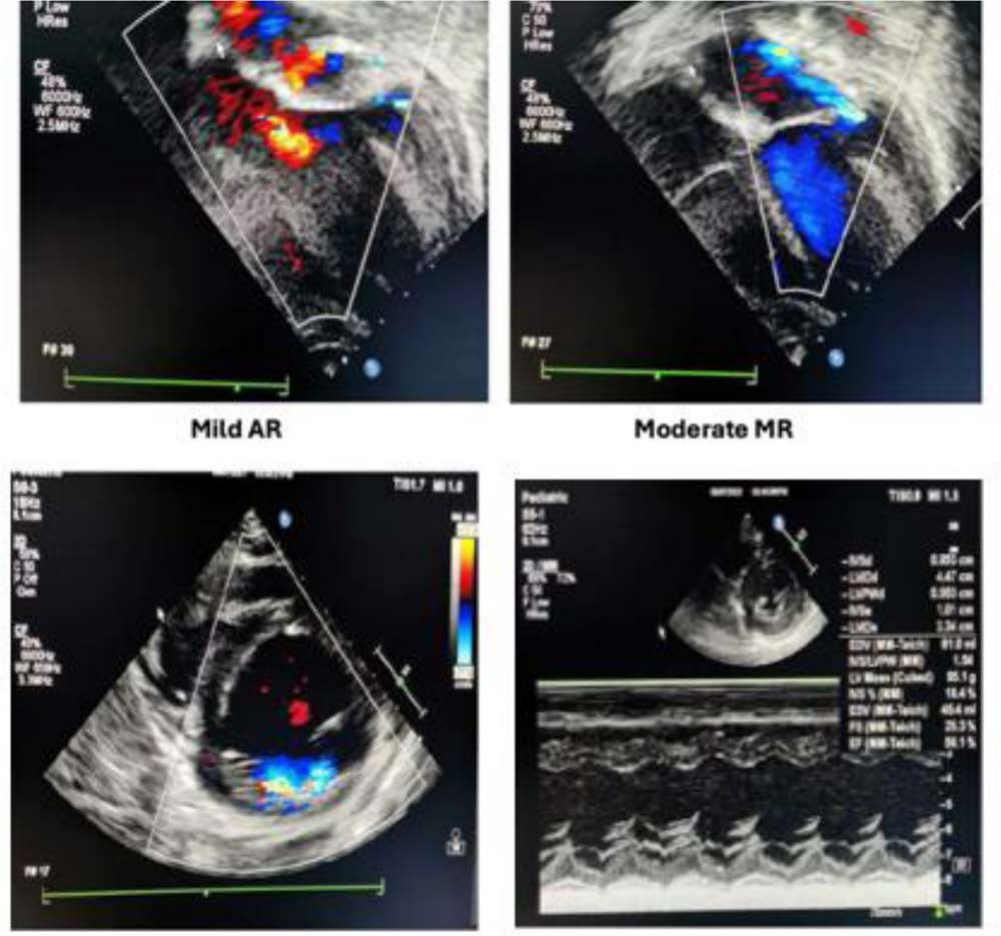
Initial echocardiogram showing mild aortic regurgitation, mild to moderate mitral regurgitation with anterior leaflet prolapse, dilated left ventricle with mitral regurgitation and left ventricular EF of 50%. EF = ejection fraction.

As shown in Fig. 2, the ECG also revealed ST-segment depression in V4 to V6 and lead II, indicating increased risk for arrhythmia and sudden cardiac arrest. Cardiac evaluations revealed a markedly elevated troponin level of 3794 ng/L (normal range < 14 ng/L), indicating significant myocardial injury. Echocardiographic assessment (Fig. 3) demonstrated mild aortic regurgitation, moderate mitral regurgitation with anterior leaflet prolapse, trace pericardial effusion, left atrial and ventricular dilation, and mildly reduced ejection fraction (EF) of 50%.

Coronary arteries were normal. These findings, consistent with pancarditis, supported a diagnosis of MIS-C. The patient fulfilled MIS-C criteria based on her age, persistent fever, close contact with a confirmed COVID-19 case, a positive SARS-CoV-2 PCR, and multiorgan involvement (gastrointestinal, cardiac, and hematological). Blood cultures were negative, and no other pathogens were isolated, fulfilling the exclusion criterion for microbial sepsis as defined by CDC and WHO. Although COVID-19 antibody testing was not performed, the positive PCR and documented exposure were sufficient to establish the required epidemiologic link.

Treatment involved intravenous immunoglobulin (IVIG) (1 g/kg/day for 2 days), IV methylprednisolone (2 mg/kg/day), diuretics, and inotropic support (transitioned from dobutamine to milrinone). Aspirin was initiated for coagulopathy resolution, as recommended by the hematology team. A 10-day course of meropenem and linezolid was completed under the guidance of the infectious disease team.

Multidisciplinary care, involving cardiology, infectious disease, hematology, surgery, and intensive care unit teams. This collaborative approach was crucial in managing the complex presentation of MIS-C and resulted in significant clinical improvement.

**Figure 4.**
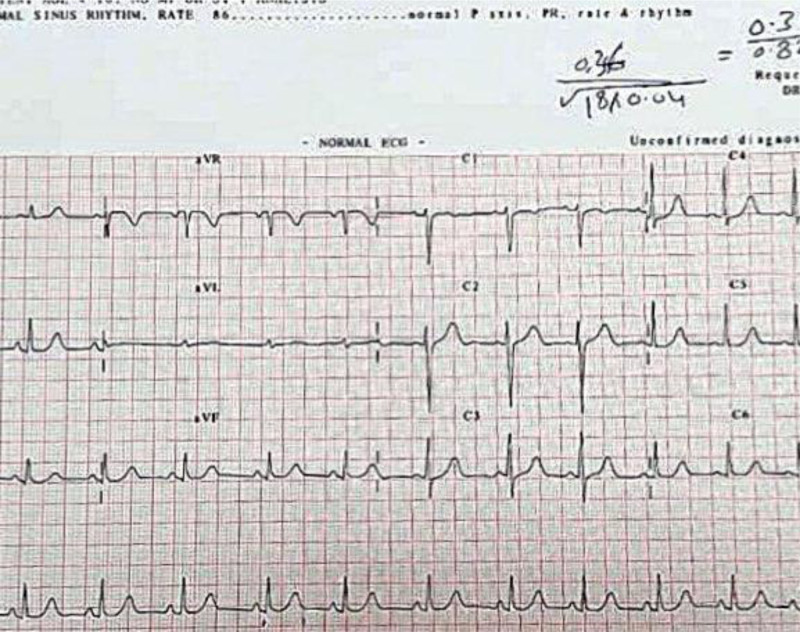
ECG obtained 72 hours after initiation of IVIG and steroid therapy, showing QTc normalization to 0.42 ms and resolved ST-segment changes. ECG = electrocardiogram, IVIG = intravenous immunoglobulin, QTc = corrected QT interval.

**Figure 5.**
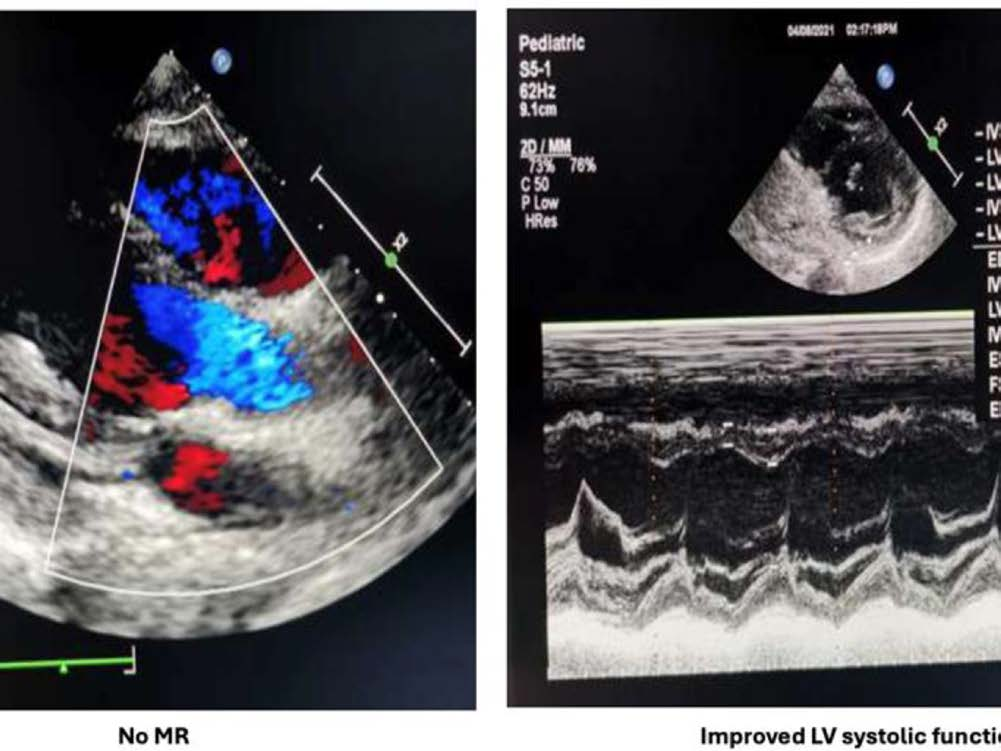
Echocardiogram obtained 14 days posttreatment showing resolution of mitral regurgitation and improved left ventricular ejection fraction (65%). EF = ejection fraction.

**Figure 6.**
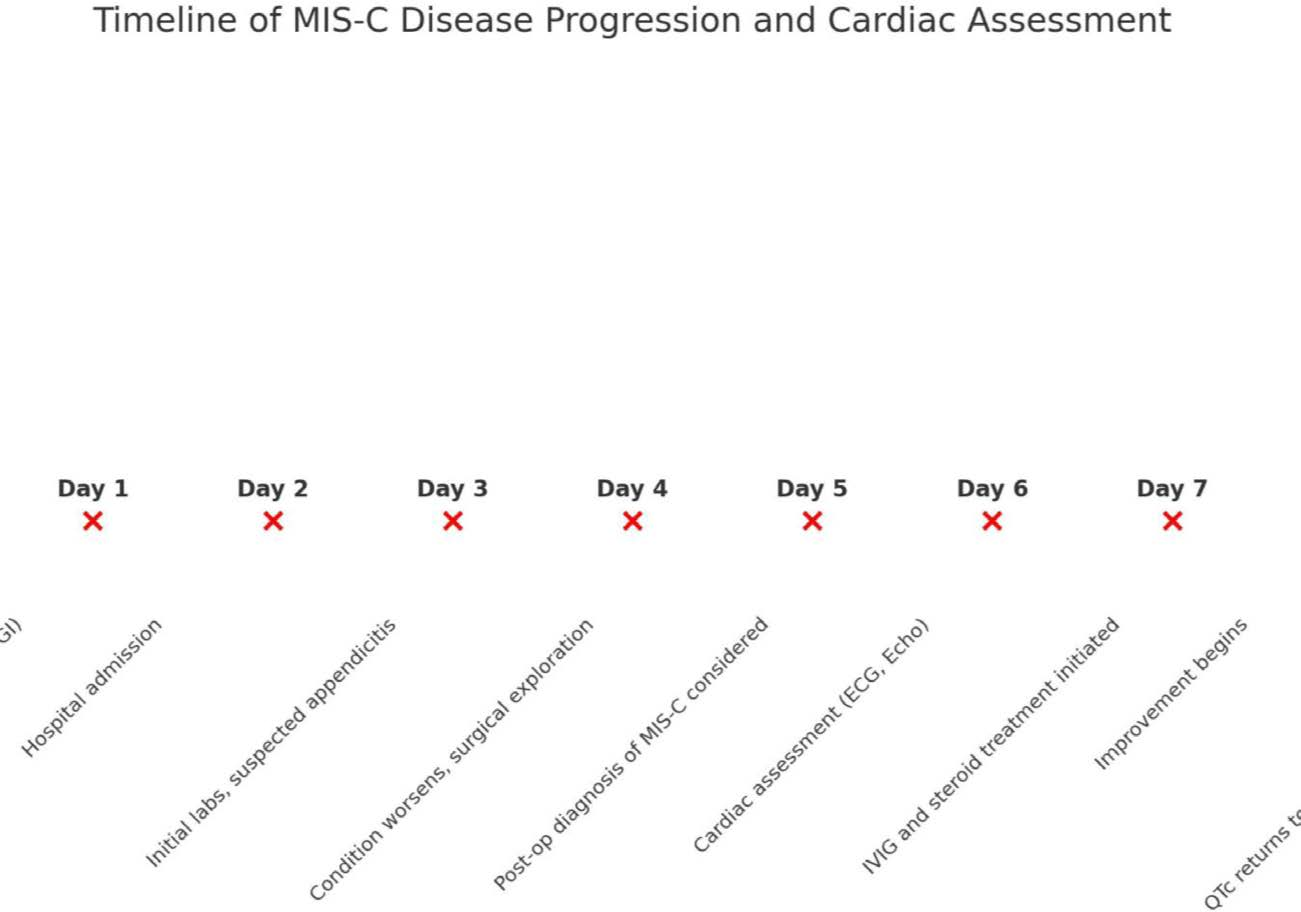
MIS-C disease progression timeline. MIS-C = multisystem inflammatory syndrome in children.

ECG normalization (QTc 0.42 ms) was observed within 72 hours after initiation of IVIG and corticosteroids (Fig. 4). Echocardiographic improvement, including normalization of EF (65.9%) and resolution of valvular regurgitation, was confirmed by Day 14 posttreatment (Fig. 5), along with normalization of troponin levels. The patient was then transitioned to the ward and subsequently discharged with scheduled outpatient cardiac follow-up. Serial evaluations at 1, 3, and 6 months, extending over a year, confirmed sustained cardiac recovery. Figure 6, summarizes the timeline of the patient’s disease course.

## 3. Discussion

The presentation of MIS-C in this 11-year-old girl, after SARS-CoV-2 infection, during the peak of COVID underscores a severe manifestation distinct from both classic COVID-19 presentations and Kawasaki disease. This distinction is critical, as MIS-C embodies a systemic inflammatory response with multiorgan involvement, a hallmark not typically observed in Kawasaki disease, which is more narrowly focused on vasculitis and mucocutaneous symptoms.^[6,11]^ Our patient’s experience, marked by severe cardiac complications including valvulitis, myocarditis, and prolonged QTc syndrome,^[6]^ mirrors the diverse clinical spectrum of MIS-C but introduces a unique constellation of symptoms that underscores the necessity for comprehensive cardiac assessment and monitoring in affected pediatric patients.^[5–7]^ The diagnostic utility of Schwartz et al’s criteria in identifying QTc abnormalities remains relevant in evaluating pediatric cardiac complications.^[14]^

The cardiac involvement in MIS-C, a predominant feature in our patient’s case, necessitates intensive care and vigilant monitoring.^[2]^ This aligns with previous findings of hyperinflammatory shock observed in children, emphasizing MIS-C’s potentially life-threatening nature.^[15]^ Previous studies indicate that a significant proportion of MIS-C patients require intensive care unit admission, with cardiac complications such as ventricular dysfunction, coronary artery dilation, or aneurysm^[16]^ being common,^[5,9,12]^ and variable severity of rhythm and electrical conduction abnormalities regardless of their baseline EF values. Coronary involvement, as seen in other reported MIS-C cases, further supports the need for long-term cardiovascular follow-up^[5,13,17]^ The co-occurrence of such complications alongside gastrointestinal symptoms that mimicked appendicitis in our patient not only complicated the initial diagnosis but also highlighted the chameleon-like presentation of MIS-C,^[18]^ which can mirror other acute conditions.^[6–8]^

Treatment strategies for MIS-C, particularly those with severe cardiac involvement, remain an area of active research and clinical debate. One intriguing aspect of MIS-C management, highlighted by the case and supported by Feketae et al, is the potential role of vitamin D. Research suggests vitamin D deficiency may exacerbate the severity of MIS-C, positing that supplementation could ameliorate the disease’s course.^[11,12]^

While our patient’s vitamin D status was not assessed, this omission points to a potential area for future clinical inquiry and management strategy refinement, considering the inflammatory nature of MIS-C and vitamin D’s known immunomodulatory effects. Recent studies from Italy have similarly emphasized the risk of cardiac dysfunction in MIS-C patients and the importance of early intervention.^[14]^ The patient was unvaccinated as well so this could relate to the severity of the presentation. Our patient’s prompt response to IVIG and glucocorticoids aligns with current therapeutic recommendations, emphasizing the role of immunomodulation in managing the syndrome.^[5,6,9]^

However, the necessity of aspirin for anticoagulation in our patient due to coagulopathy illuminates the multifaceted approach needed for these complex cases, where cardiovascular complications are at the forefront.^[2]^

One additional consideration in MIS-C management is the role of vitamin D. Feketea et al^[7]^ and others^[8]^ have suggested that vitamin D deficiency may exacerbate the severity of MIS-C, and that supplementation could improve clinical outcomes.^[19]^ Although our patient’s vitamin D status was not assessed, this represents a potential area for future clinical investigation, particularly given the immunomodulatory effects of vitamin D.

The absence of a need for intensified therapy in our case, despite its severity, add an interesting dimension to the ongoing discussion about the optimal management strategies for MIS-C, suggesting that conventional treatments can be highly effective when applied judiciously and tailored to the patient’s specific clinical profile.

To conclude our analysis, this case underscores the critical nuances of diagnosing and managing MIS-C. Given the evolving clinical landscape in 2025, this report also highlights how MIS-C should still be considered in the differential diagnosis of pediatric patients presenting with multisystem inflammatory symptoms, particularly when there is a recent history of COVID-19 exposure or infection. Clinicians must remain vigilant as the post-viral inflammatory spectrum continues to evolve, a syndrome that remains a complex puzzle in the COVID-19 landscape. These observations are in line with the CDC’s updated clinical guidance for healthcare providers managing MIS-C.^[9]^ Our findings highlight the syndrome’s potential for severe cardiac complications, reinforcing the need for a tailored, patient-specific approach to treatment. The responsiveness to IVIG and glucocorticoids, observed in our patient, making Successful outcomes for one of Saudi Arabia’s early severe pediatric MIS-C cases post-COVID-19 and provides valuable insight into the potential efficacy of current therapeutic strategies,^[5,6,9]^ yet leaves open questions regarding the optimization of treatment protocols. These observations are in line with the CDC’s updated clinical guidance for healthcare providers managing MIS-C.^[20]^

## 4. Conclusion

This case report delineates a pediatric instance of MIS-C that aligns with the criteria established by major health institutions. It underscores the imperative for clinicians to maintain heightened vigilance for potential MIS-C among pediatric COVID-19 patients, as early recognition and timely intervention are paramount for improving outcomes.

Our findings highlight the importance of rapid intervention, especially considering the severe cardiac manifestations (including QTc prolongation, myocarditis, and pancarditis) observed in this case. QTc prolongation is a marker of cardiac electrical instability and places affected patients at risk for life-threatening arrhythmias such as torsades de pointes. As demonstrated in this case, it demands close telemetry surveillance and serial cardiac assessments.^[10]^ The patient’s clinical improvement and sustained recovery validate the importance of early immunomodulatory therapy in mitigating cardiac complications. Successful management involved a multidisciplinary team comprising cardiology, hematology, infectious disease, pediatric surgery, and intensive care specialists, reinforcing the potential for favorable outcomes even in severe MIS-C presentations.

However, the factors contributing to the rapid progression and exacerbation of MIS-C remain incompletely understood, highlighting a gap in current understanding and an area ripe for further investigation. The collective sharing of clinical experiences and case studies^[11]^ remains invaluable, fostering refinements in clinical approaches and deepening our comprehension of MIS-C’s evolving presentation.

Furthermore, the exploration into the long-term sequelae of MIS-C is an emerging field. As we continue to navigate this postinfectious syndrome, future studies must focus on the lasting cardiac and systemic impacts^[12]^ to guide comprehensive patient care strategies beyond the acute phase.

## 5. Limitations

Limitations of this report is that it reflects a single patient’s experience, which may not represent the entire spectrum of MIS-C presentations. Additionally, vitamin D levels were not evaluated, which could have provided insight into potential contributors to disease severity. Our comprehensive follow-up indicated full cardiac recovery without long-term complications, demonstrating the effectiveness of early multidisciplinary intervention in pediatric MIS-C cases.

## Author contributions

**Conceptualization:** Sultan Alharthi.

**Data curation:** Sultan Alharthi, Suzan Abdullah Jafri, Mazen A. Alzaedi, Nejood Metaeb Aljaed, Mohammed Mahmoud Eldoskey, Mohammed Oshi, Naglaa Mohamed Kamal.

**Investigation:** Sultan Alharthi, Mohammed Mahmoud Eldoskey.

**Methodology:** Sultan Alharthi, Mohammed Mahmoud Eldoskey.

**Project administration:** Sultan Alharthi.

**Supervision:** Sultan Alharthi.

**Validation:** Sultan Alharthi.

**Visualization:** Sultan Alharthi.

**Writing – original draft:** Sultan Alharthi, Suzan Abdullah Jafri, Mazen A. Alzaedi, Nejood Metaeb Aljaed, Mohammed Mahmoud Eldoskey, Salma A.S. Abosabie, Mohammed Oshi, Sara A. Abosabie, Naglaa Mohamed Kamal.

**Writing – review & editing:** Sultan Alharthi, Salma A.S. Abosabie, Sara A. Abosabie, Naglaa Mohamed Kamal.
